# Book Reviews

**Published:** 1829-02

**Authors:** 


### CRITICAL ANALYSES.

Quæ laudanda forent, et quæ culpanda, vicissim

Illa, prius, cretâ; mox hæc, carbone, *notamus*.—Persius.

*A Treatise on the Nature and Cure of Intestinal Worms of the Human Body: arranged according to the Classification of* Rudolphi *and* Bremser, *and containing the most approved Methods of Treatment, as practised in this Country and on the Continent.* By William Rhind, Surgeon, Member of the Royal Medical Society of Edinburgh. *Illustrated by six Plates.* —8vo. pp. 152. London: S. Highley, 1829.

Whoever has had opportunities of becoming acquainted with popular, and perhaps even professional, prejudices respecting the cause of various ailments, especially of children, must, we apprehend, be convinced that the presence of worms in the alimentary canal is not unfrequently presumed upon very slender grounds. If, in infants of a very tender age, obscure symptoms of general disturbance arise, they are commonly attributed to teething. At a more advanced period, when obscurity hangs over the complaint, "worms" are a convenient resource when no other explanation can be offered, and many a hapless child is drugged for months to remove a cause which in reality does not exist. In making this observation, we would not have it inferred that we are unconscious of the many and severe ailments that arise from the existence of these troublesome parasites. We would wish to invite a stricter attention to the subject, by which the practical errors which we have hinted at may at least be rendered less common, if they cannot be entirely removed.

In this country the subject of intestinal worms has been much neglected. Dr. Hooper published, in the year 1799,* an interesting paper on the five species of worms which are found within the intestinal canal of the human body. He confines himself, however, entirely to a description of the external appearance and anatomical structure of these, without giving any information regarding their history, symptoms, and method of cure, or at all mentioning the different species of worms which inhabit the other cavities and textures of the body. Dr. T. Bradly has added but little to the subject. Dr. Chamberlain wrote expressly for the purpose of recommending a particular medicine for the cure of tænia, &c. in the Stizolobium, or cowhage, and does not enter upon a general description of worms. Possessing, then, hitherto, such meagre and unsatisfactory information upon the subject, it appeared to the author of this Essay that a work on the nature and treatment of intestinal worms was yet a desideratum in this country, and to supply this want the present Treatise has been attempted.

Mr. Rhind has adhered to the classification and specific descriptions of Rudolphi, and from the work of Dr. Bremser he has culled much useful and appropriate information. He is also occasionally indebted to Dr. Hooper for his anatomical descriptions. The most approved practice of this country is given, together with a view of the mode of cure adopted by Bremser. The drawings have been executed by Captain T. Brown, F.R.S.E., whose knowledge of natural history is said to enable him to delineate the different objects with more fidelity than could be expected from a mere copyist.

*Of the formation of worms in the intestines.*—Such is the disposition in nature for the support of animal existence, under every variety of circumstance, and in every possible situation, that all animals, even down to very minute species, have other animals, still smaller, which inhabit their bodies, and derive their nourishment, and live, and propagate their species, in their various textures.

"Of these parasitical animals which are found among the various classes of the animal kingdom, Rudolphi enumerates 1100 different species. Some of these worms are common to several classes of animals, but others again are peculiar to and only found in one particular species.

"The Ascaris lumbricoides, or large round worm of the human

* Memoirs of the London Medical Society, vol. v.

species, is to be met with also among pigs, horses, and cows; whereas the two species of tapeworm found in the human body are distinct from those of all other animals.*

"Every different structure and cavity of animal bodies will be found liable to be tenanted by these animals; and, for the most part, to be exclusively inhabited by a particular species. There have been worms found in the brain, in the lungs, in the liver, the biliary ducts, and even in the heart itself; and Hopkinson and Morgan discovered a species of worm (the Filaria papillosa) in the anterior chamber of a horse's eye. We find, also, in the tenth volume of the Transactions of the Royal Society, another worm, which is described by Captain Brown as a new species, the Ascaris pellucidus, which also inhabits the eyes of horses in India, and may be seen swimming about in the aqueous humor with great activity.

"It sometimes happens that the eggs and larvæ of various insects get introduced into the body, and are there developed; but these are not to be confounded with the animals which are peculiar to, and exist and propagate their species in, the cavities of the human body. It is of these latter that a particular description is proposed to be given in the following pages.

"That the intestinal worms of the human body are of a peculiar kind, and different from any which are found to exist in the earth or water, is sufficiently evident from their distinct and peculiar formation, from their living and propagating their species in the body, and from their incapability of sustaining life for any length of time if removed out of it. These worms, when exposed to cold air or water, very quickly die; whereas, had they previously existed in the earth or water, the change could not have so completely affected them.

"If they were not distinct worms, but come from without, why not also inhabit the same parts of the body promiscuously? Whereas it will be found that some of the species live in the small intestines, and others, again, always in the large." (P. 13.)

Supposing it to be true that the intestinal worms of the human body are different in appearance from any which are found to exist in the earth or water, it does not follow they are "of a peculiar kind." For it is well known that considerable alteration of structure will result from change of food and habitation, in worms or the larvæ of insects introduced into the human intestines from without. A difference of food, for instance, alone produces a growth and development of sexual organs in the honey bee, and converts what have hitherto been called neuters, but which are really imperfect females, into queens, or bearing bees.† It

* Bremser.

† Good's Study of Medicine, vol. i. p. 309.

may be also true that worms which have been long resident in the intestines, "when exposed to cold air or water, very quickly die," and still they may have previously existed in the earth or water. Such a change may have occurred in the constitution of these animals, from the circumstances just referred to, that they are no longer capable of bearing the effects of those external agents, amidst which they were by nature destined to live. But, in fact, Linnæus himself pointed out that the Tænia solium exists, though much smaller, in muddy springs. Menander also, cited by Rosen, Unzer, and Tissot, declares that he has found in water the same species of worms that inhabit the human body!* Dr. Bremser is of opinion that the origin of worms in every body is at first by a *primitive* or *spontaneous formation*; the *bildungstrieb* of many German physiologists. This hypothesis is opposed by Mr. Rhind, and we think upon very solid grounds. Such a doctrine is unsupported by a single fact. It is contrary to all analogy drawn from the animal kingdom: for in no other class of animals is there an instance of spontaneous formation.† We believe it is generally admitted that "a certain state of the system and bowels is necessary to favor the production of intestinal worms, and that a healthy state of the bowels is sufficient to resist them, even should they be introduced either alive or in the state of eggs." Pallas has demonstrated by experiment that worms may be propagated by the injection of their eggs into the body.

"By a small incision, he introduced into the abdominal cavity of a dog the eggs of a tænia from another dog; and, after the expiration of a month, he found young tæniæ in the cavity. In this case, not being within the intestine, they were not liable to be expelled by the healthy action of the bowels; and the natural warmth and moisture of the abdomen favored their production." (P. 22.)

In many cases it may doubtless be very difficult to determine in what manner worms are produced in the intestines or other parts of the animal body. Upon this subject Mr. Rhind offers several suggestions, which are very similar to the opinions given by Good in his Study of Medicine (vol. i. p. 306.) Without having recourse to the doctrine of *equivocal* generation, it must be presumed that the origin of

* Dict. des Sciences Med. tome 57, p. 213.

† It was originally supposed by Aristotle that, during the process of putrefaction in animal fluids, worms were spontaneously formed. This opinion has been maintained by some modern authors, amongst others Needham, who was ridiculed by Voltaire for having asserted that eels were created in mutton gravy!—Rev.

worms is *ab externo*. As a proof of the extrinsic origin of the Ascarides vermiculares, we may mention the following fact recorded by Dr. T. M. Barry:* "In the year 1797, a family residing near the town of Macromp, in Ireland, suffered severely from this species of worm; and, upon examination, it was found that a spring, from which they constantly drank, was infested with worms which corresponded in appearance with the ascarides."

The opinions of the author as to the causes of the formation of worms are in unison with those commonly maintained. A general laxity and debility of the whole system, but more especially a feebleness of the intestines, is the disposition of body which is most prone to worm affections.

"A want of due harmony, too, between the several parts of the alimentary system, an imperfect digestion of the food, and a deficiency of the various juices necessary for converting this food into nourishment, or an overactive digestion, producing more alimentary matter than the absorbent vessels can take up, are both equally favorable to the production of worms.

"When the nutritious matter taken into the stomach is imperfectly digested,—when there is a deficiency of the necessary fluids for this important purpose,—and more especially when there exist a feebleness and torpidity of the stomach and alimentary canal, the imperfectly digested chyle accumulates in the bowels, passes into a state of fermentation, gives rise to an undue quantity of mucous matter, and affords a favorable opportunity for the development of the various worms which feed on the chyle, and find an easy lodgment in the bowels, from their impaired action and diminished peristaltic power. On the other hand, when the digestive powers are over vigorous, when a greater quantity of nutritious matter is prepared by the active state of the stomach than the absorbent vessels of the system can take up, this alimentary matter accumulates on the internal coats of the intestines, and thus becomes favorable for the production of worms. It is from this cause that we occasionally find robust and healthy people affected with this disease: and this constitutional temperament, or predisposition to this disease, may be often transmitted from one person to his descendants; thus exemplifying the hereditary tendency to worms which writers have remarked." (P. 27.)

It is well known that certain kinds of diet have an effect in predisposing the body to worm diseases; such as crude raw vegetables, unripe fruits, various sweetmeats, &c. Salt, from its stimulating qualities, is known to be a preventive of worms. Lord Somerville, in his address to the Board of Agriculture, relates the following circumstance:

* Transactions of the Royal Irish College, vol. ii.

"The ancient laws of Holland ordained men to be kept on bread alone, *unmixed with salt*, as the severest punishment that could be inflicted upon them in their moist climate. The effect was horrible: these wretched criminals are said to have been devoured by worms engendered in their own stomachs." Salt, too, when given to graminivorous animals, besides its other beneficial effects as a stimulant, is of advantage in causing the destruction of the various intestinal worms to which this class of animals are liable. For this purpose it has also been used as a remedy for sheep with diseased livers; which disease is frequently caused by the lodgment of a peculiar worm in that viscus." (P. 33.)

Spirit drinkers have been found less liable to worms. They have been expelled from the intestines by using alcoholic liquors as a remedy.

Mr. Rhind gives a very accurate anatomical description of the various species of worms that infest the human body. For this part of the subject we must refer our readers to the work itself. An abstract from it would be but of little service without the plates, which illustrate the peculiar formation of each kind of worm.

"*Symptoms attending the presence of worms:*

"The appearance of the countenance is changed, it is generally very pale or of a leaden colour, with a red circumscribed spot in one or both cheeks. The eyes lose their brilliancy, the pupil is enlarged, and a blue rim is perceivable round the under eyelid. The nose is swelled, and very generally the upper lip is somewhat tumified, and there is a continual itching and irritation in both these. Sometimes, too, there is a bleeding from the nose. There is also headach, throbbing in the ears, a foul tongue, more saliva than natural in the mouth, and the breath is very fetid, especially in the morning. The appetite is variable: sometimes it is quite gone, and at other times it is voracious, with a continual gnawing sensation at the stomach. There is also nausea, and a desire to vomit: when this takes place, the fluid ejected is limpid like water. There are often violent gripings, and these are principally felt about the umbilical region. The alvine excretions are glairy, and sometimes tinged with blood. The urine is turbid, and, after it has deposited a sediment, it has the appearance of milk-and-water. The belly, too, is hard, and has a feel like a drum. There is a general emaciation of the body; the sleep is troubled, accompanied by grinding of the teeth. The patient is generally lazy and indolent; sometimes in good and sometimes in irritable temper. Blindness, deafness, delirium, even apoplectic and epileptic fits, have been known to have their origin from worms. The last and most decisive symptom observed is, that in the matter vomited, but more generally in the alvine excretions, entire worms or portions of them are perceived.

"It must be remarked, that all the above symptoms are not always found in the same individual; nor do any of them, except the last, exclusively indicate the presence of worms. One or more of these symptoms may be indications of the existence of several other diseases, as water of the head and some others; but when these symptoms occur, and cannot be attributed to any other cause, the strong presumption is that this cause is worms. At the same time it may be mentioned, that worms sometimes exist, and that in considerable quantities, without causing any inconvenience or any bad symptoms whatever." (P. 105.)

It must not be too hastily concluded, because worms, or portions of worms, are voided during the existence of disease, that the malady has been caused by their presence. They may have previously existed in the intestines, and, from the altered state of the body, or the effect of medicines, may have been expelled.

"It is in this way that the notion of worm epidemics and fevers must have originated. Fever, when it seizes a patient, generally proves the death of the worms contained in the body: and this circumstance occurring in those in whom worms had previously existed, had very naturally given rise to the erroneous conclusion that these worms were the cause, and not the effect, of the fever." (P. 108.)

Much difference of opinion has existed as to the fact whether worms perforate the coats of the stomach and intestines. Rudolphi and Bremser are of opinion that they do not. There are many cases on record, however, which prove the contrary.*

We now come to the most interesting, but unfortunately, in the present state of our knowledge, not the most satisfactory, part of the subject, *the method of cure.*

Our object is twofold: first, to destroy and expel the worms; second, to correct that particular state of the general system, and especially the intestinal canal, which has been the cause of their formation. The author is of opinion that those medicines which are given with a view of destroying intestinal worms by their mechanical action, are of very doubtful operation, and in all probability owe the whole of their good effects to the powerful purgatives with which they are either conjoined or immediately followed.

"Even the cowhage (Stizolobium), a remedy so much recommended by Chamberlaine, and which for a considerable time was in much vogue for the cure of tænia, though calculated to act as

* Cases in which Lumbrici were evacuated by Ulceration through the Parietes of the Abdomen. By W. Young, M.D. (Glasgow Med. Journal, Nov. 1828.)—Rev.

the most powerful mechanical agent, from the peculiarly sharp, penetrating, and minute spiculi of which the down of the pods is composed, has never been found effectual, unless purgatives are used at the same time." (P. 113.)

Of the cure of the maw, or threadworm, (Oxyuris vermicularis,) and long threadworm, (Trichocephalus dispar.) —These two species are found in the large intestines, or lowest part of the intestinal canal. Sometimes they cause little or no inconvenience. More frequently they produce great irritation, heat and pain about the anus, especially after exercise. They are most common in children, and sometimes cause convulsions.

"Those medicines given by the mouth, with a view to their destruction, generally, in the course of the long passage through the intestines, lose their peculiar virtues, and become of little use: injections, therefore, are most to be depended on. Aloes, however, are known to have the property of acting particularly on the rectum and cœcum, and of passing through the other small intestines little changed: from two, three, to six grains of aloes, given in a pill or powder every morning, often destroys these worms in considerable numbers."* (P. 115.)

Dr. Bremser's mode of cure consists in the exhibition of purgatives and bitters. The formulæ of his prescriptions are given. Dr. B. has found the irritation relieved, and the worms destroyed, by an injection of any of the common oils. In obstinate cases he advises the fumes of tobacco, or an enema of the infusion of the male fern.

"As these worms most commonly affect young children, it is of great consequence to have the medicines exhibited in as small bulk as possible. The following can be given disguised in a little jelly, &c. ℞. Pulv. Aloes, gr. xvi.; Pulv. Scammoniæ gr. viij.; Sacch. Alb. ℈i. Misce. To be divided into four or eight powders, according to the age of the child; one powder every morning.

"The following injection is then to be given: ℞. Ol. Terebinth. ʒij.; Ol. Olivar. ℥ij. Misce, pro enema. Or, ℞. Pulv. Aloes ʒss. To be dissolved in a little milk or gruel for an injection.

"The quantities in both the above to be doubled, if necessary, according to age, &c.

"The infusion of tobacco, also, in the proportion of ʒi. of the leaves to 1℔. boiling water, letting it stand ten minutes, is also a powerful enema." (P. 117.)

* It is well known that worms are very partial to milk. And for the purpose of effecting the expulsion of those species which inhabit the rectum, it has been proposed to place the patient in a hipbath of milk, by which they may be allured from their abode. This plan has sometimes succeeded even in cases of tænia. (Dict. des Sc. Med. tome 57, p. 199.)—Rev.

Sometimes these worms escape from the anus, and creep into the vagina, causing great irritation. Injections of equal parts of cold water and vinegar, repeated frequently, will be found to destroy them.

Of the cure of the long round worm (Ascaris lumbricoides).—These worms are found in the small intestines, and feed on the pure chyle. They sometimes exist in adults.

"These worms are generally easily expelled; but, to ensure this completely, as also the destruction of their eggs, it is proper to persevere with the vermifuge medicines for some considerable time, and to keep up a continued action in the intestinal canal. A combination of medicines, too, which act on every part of the alimentary canal in succession, will be found the most complete and efficacious." (P. 120.)

Purgatives, succeeded by tonics, are required. To prevent future attacks, the diet and state of the digestive system must be strictly attended to.

Of the cure of the Bothriocephalus and Tænia, or tapeworm.—To dislodge these worms is a task of great difficulty. They frequently cause considerable mischief. Dr. Bremser tells us that he has treated more than 500 persons of different ages, affected with tapeworm, with uniform success. None of his patients, after going through the proper course of medicines, having had occasion to apply to him again. He commences by giving the same electuary he recommends for the other species, for several mornings in succession. We subjoin the formula:

"℞. Sem. Cinæ Tanacet. Rud. Contus. ℥ss.; Pulv. Valerian. ʒij.; Pulv. Jalapæ ʒiss. ʒij.; Sulph. Sodæ ʒiss. ʒij.; Oxymel Scillæ q. s. ūt ft. electuarium. Dose, two or three teaspoonsful in a morning."

After some time he gives the empyreumatic oil of Chabert, which is composed of one part of empyreumatic animal oil, and three parts of oil of turpentine. The dose is two teaspoonsful in a little water, morning and evening: it may be increased or diminished according to its effects. "If this medicine should affect the bladder, an emulsion of oil, mucilage, or other bland liquid, is to be taken frequently, to correct the disagreeable symptoms." Having persevered in this course for ten or twelve days, purgatives are to be exhibited. Jalap, senna, and soda, are recommended in combination. Four or five ounces of the oil are generally sufficient to effect a cure; or, in obstinate cases, six or seven ounces. The medicine must be continued some time to ensure the complete eradication, not only of the worms, but of their eggs. If there is a disposition to form glairy matter in the intestines, a tonic medicine is given, consisting of compound tincture of aloes, tincture of muriate of iron, and elixir of vitriol. Dr. B. is aware that this prescription is unchemical: experience, however, has proved its efficacy. He restricts the patients to no particular regimen, except forbidding them the use of dry leguminous substances, too much farinaceous diet, and all substances of an oily and fatty nature. He considers the empyreumatic oil of Chabert an effectual cure for worms, especially tænia, so much so as to supersede the use of all other remedies. Rudolphi also bears testimony to its success. As it is apt to produce nausea, griping, and strangury, the dose should be small at first, and gradually increased.

Mr. Rhind thinks, and we believe correctly, that the oil of turpentine is the active ingredient in this medicine.

"The oil of turpentine, when taken alone, is very apt to pass off by the urinary vessels, and to affect the neck of the bladder, thereby causing great irritation, and often strangury. By being conjoined with the castor oil, it more readily passes off by the bowels; and, exhibited in this manner, is a sure and efficacious remedy against the most obstinate cases of tænia, and may also be given for the expulsion of the round worm and small threadworm: for the latter, either by the mouth or, what is far better, in the form of an enema. During the exhibition of the medicine, the patient should drink copiously of bland broths, such as beef-tea, &c.; and, if there is any irritation in the bladder, the free use of an infusion of linseed will be found to allay the uneasiness and pain. Some patients cannot bear more than from twenty to thirty drops of the oil of turpentine, while others can take one to two drachms with impunity: the dose, therefore, should be cautiously regulated at first, so as not to frighten or disgust the patient." (P. 129.)

When all traces of the worms have disappeared, we must endeavour to prevent their future formation, by attending to the second indication, that of strengthening the system, and the bowels in particular, by tonic remedies, such as the sulphate of quinine or carbonate of iron.

*Of the larvæ of insects and other animals found in the human stomach and intestines.*—The larvæ of the various species of insects, as well as other worms and reptiles, often find their way into the human body, and are afterwards voided by the mouth or per anum, in a living or perfect state. Mr. Rhind observes, that these animals apparently have access to air in the stomach and intestines; for many species are voided alive, to whose existence respiration in some form is absolutely necessary.

In the fourth volume of the Transactions of the King and Queen's College in Ireland, a singular case is recorded by Dr. Pickells, of a young woman who discharged, at different times, an immense quantity of insects from her stomach, chiefly of the beetle tribe. The author has repeatedly seen the larvæ of the various species of moths, of the common flesh-fly, &c. voided from the bowels.

The following case was communicated to Mr. Rhind by Mr. A. Anderson, surgeon, Haddington.

"Robert Dixon, farm servant, Markle, Haddingtonshire, was, in the summer of 1826, engaged in driving lime to the fields, and was in the habit of frequently drinking from the ditches on the roadside. In the end of the same year his disorder commenced with an increased desire for food, a vomiting up of fetid slimy matter from his stomach, which made him cough, and with which he was attacked two or three times a day: nearly half a pint would come up at a time, accompanied with sour belchings and eructations, and a most obstinate state of bowels, five or six days sometimes intervening without a stool. He felt a swelling and fulness of the right superior portion of the stomach, which was very painful when pressed. Slept very well, except on his right side; for, when he attempted to lie on it, an almost continued working up of the slimy matter took place, which made him sit up, and brought on cough.

"He continued in this state till June 1828, using a variety of medicines, and undergoing a variety of medical treatment, without any relief.

"On the 17th June, Mr. Anderson was consulted, and ordered him a strong solution of carbonate of soda, and pills of calomel, hyoscyamus, and extract of gentian. On the second day after the exhibition of these medicines, in one of his severe fits of vomiting, he ejected from the stomach an animal of about four inches in length, which proved to be the common species of gray snail, (the Limax major.) It was quite lively and vigorous when voided, and lived in Mr. Anderson's possession for five days afterwards. After this the patient's distressing symptoms of vomiting, &c. disappeared, and he is now (10th October) about to resume his usual occupation." (P. 140.)

It would be but an equivocal compliment to say that Mr. Rhind's treatise contains more information than any previous work upon intestinal worms, after having stated that hitherto this subject has been much neglected in this country. The practical inquirer will find in this essay a satisfactory account of all that is known upon the subject.

Notwithstanding the confidence with which Dr. Bremser speaks of the efficacy of the treatment he adopts, we fear that Dr. Mason Good's observation will still apply, "that a decisive vermifuge process is yet a desideratum in medical practice;" such, at least, is the result from our own experience, and our practice has not materially differed from that of Dr. Bremser, unless, indeed, the "huile empyreumatique" of Chabert possesses greater anthelmintic powers than the oil of turpentine, which we do not presume to be the case.

---

*A Letter addressed to his Excellency the Right Honourable General the Earl of Chatham, K.G. Governor of Gibraltar, &c. &c. &c. relative to the Febrile Distempers of that Garrison.* By W. W. Fraser, Esq. Inspector of Hospitals, and Medical Superintendent of Quarantine at Gibraltar.—8vo. pp. 49. Callow and Wilson, 1826.

When every arrival brings accounts of the progress and increasing mortality of the fever at Gibraltar,* and when we recollect the great loss of life formerly occasioned there by, it is presumed, similar fevers, coupled with the difference of opinion which then existed, regarding its nature and treatment, we are led to look into the accounts of former periods, that we may form some general opinion as to the probable extension and results of the present visitation. For this purpose, we reasonably expected precise and satisfactory information from the last publication on the subject, a pamphlet by Mr. Fraser, considering the peculiar advantages enjoyed by that gentleman, for obtaining the best means of judging fully and accurately. In this expectation, however, we may as well state at the outset, we have been disappointed. Mr. Fraser, it appears, has long resided at Gibraltar, and was, for years, at the head of the medical staff, and medical superintendent of quarantine there.

The publication in question is in the form of a letter to the governor of the garrison; but is clear that some further object was contemplated, and that it was likewise meant to communicate knowledge to the professional reader, else it should not have been encumbered with doctrines and directions which could not interest the governor, and will not, we fear, instruct those who are eager for knowledge on this interesting subject. The letter to the governor will not, we are sorry to say, dispel the darkness

* More favorable accounts have happily been received since this article was written.—Editors.

which rested on the subject, nor settle the disputes which have arisen regarding it. It is almost in every way unsatisfactory: obscure in manner, confused in statement, verbose and affected in style, while pretending to be terse and epigrammatic; there is, indeed, little to defray the trouble of reviewing it; and, were it not for the present condition of Gibraltar, and some dangerous dogmas which it contains, we should certainly have never written a line regarding it.

In a pamphlet of forty-nine duodecimo pages, Mr. Fraser has given us opinions in a great variety of important subjects, but in a manner which renders it impossible to follow him, without writing at much greater length than he has thought proper to do; his notions are given not only without order, but in such total defiance of order, that it would be an easier matter to render an intelligible account of Good's Study of Medicine than of this tiny production. We shall, therefore, for our own satisfaction, and the reader's ease, glance at the principal points named, not fully stated, much less argued, in the following order: first, the origin of the disease; second, its nature; third, its treatment.

With regard to the first point, it is difficult to ascertain Mr. Fraser's opinion, from the strangeness of some of the terms which he employs, and the confusion into which he has contrived to throw those in general and well-defined use. Thus he appears, at one time, to employ the words infection and contagion as convertible terms; and, at another, to apply them in the sense for which they are at present generally used, respectively. Then we are told of "epidemic influence," and "epidemic constitution," as explanatory of all difficulties; again, we are informed that "it is obviously infectious." From these and similar modes of expression, so palpably obscure, it is impossible to say what Mr. Fraser's notions on the subject of contagion certainly are; whether he regards it as an inherent and essential quality of the disease, and therefore capable of distant transportation, or as an accidental adjunct acquired during the progress of the disease. But, from the general tenor of the pamphlet, the bustle about seclusion and quarantine, the faith with which he seems to repose on the doctrines of his friend Mr. Pym, and the danger which would be incurred by the importation of typhus, put forth as a parallel case, we are led to infer that the former is his opinion. Now we do not intend to enter on the question of the origin of the fever in 1813, because we have neither time nor space for such an inquiry, and because, after all that has been written on the subject, what we could say might not be received with much patience or good will; but we confidently refer to the writings of Dr. Burnett, for clear and, as it appears to us, conclusive facts and arguments against the belief that it was then a disease foreign to the soil of Gibraltar. It has been objected that Dr. Burnett had not such ample means of forming correct opinions on the subject, as those gentlemen who had been long resident on the rock of Gibraltar. We are aware of the importance of extended opportunities of judging, and allow them their full weight; but we would hint to the objectors, that opportunities are not always properly cultivated, that experience and observation are not always the same thing, that moral vision is not equally clear in all persons, and that one man can see at a glance what another shall not perceive during a long life. We will only add, our firm conviction, founded on no trivial grounds, that such fevers are always the offspring of the earth, or its inorganic tenants, on which they appear; and that they are no more capable of being carried from Bulam to the West Indies, and thence to Gibraltar, than they are to be transported from the last place to Greenland. It is not a little curious and mortifying to observe, in proof of the power which prejudice and partisanship possess to pervert reason and warp judgment, that while such labour and pains are taken to trace the epidemic fevers of Gibraltar to a foreign source, no difficulty is found in accounting for the arrest of contagion on the neutral ground. How can it be reconciled with the most simple proofs of reasoning, nay with the plainest dictates of common sense, that the germ of a disease which could be carried 4000 miles to Gibraltar, could render the place desolate in a few weeks, and while the inhabitants within the walls were yet falling its victims in great numbers daily, when the garrison must, in fact, have been a vast magazine of contagious material; if contagion was the cause of the mortality, how, in the name of common sense, could such things happen, and the disease during the whole period never reach the neutral ground?

While multitudes were perishing within the walls, their friends, the inmates of the same sheds, and the partners of the same beds, were removed to the neutral ground, yet they did not carry the disease with them, or, if they did, had not the power of communicating it to any one there; nay, patients were carried from the town to the neutral ground labouring under black vomit, and in the act of dying, yet the disease was not propagated. How can such things be, if the fever is endowed with contagious properties? Surely such plain, prominent, incontrovertible facts ought to open the eyes of every man who is not determined to keep them shut; and, to remove every reasonable apprehension regarding the importation of this disease, we are persuaded that the medical superintendent of quarantine, and similar functionaries at Gibraltar, will do wisely to look closely at home for the cause of their epidemic fevers, instead of turning their eyes and inquiries to Siam, Bulam, or Havanna; that they should throw off their fears of foreign invasion, and try to defend themselves against a climatic enemy; and that, if they seek carefully and skilfully, they will find within their own walls, and in their own houses, abundant cause for all their suffering from epidemic fever.

Secondly. Of the nature of this fever we shall only say a very few words, as we hold opinions on the subject, in some measure peculiar, and as this is not the place, if there were opportunity, to set them forth; but we think it right to state why we cannot agree with Mr. Fraser in one or two particulars.

Respecting the often agitated question of second attack, Mr. Fraser agrees with Mr. Pym, who gave himself much trouble to secure to himself the honour of discovering what does not exist. That those who have once had the fever in question, the Bulam of Pym, the epidemic fever of Mr. Fraser, (a good latetudinarian name, by the by,) are not very susceptible of the disease, for a considerable time at least, we believe, and indeed have the means of knowing; but that one attack constitutes any thing like perfect immunity from another, it is now too late to maintain. For proofs on this point, it is enough to refer to Dr. Burnett's work on Mediterranean Fever, as far as Gibraltar is concerned; and, if the epidemic fevers there be of the same nature as the concentrated fever of the West Indies, a cloud of witnesses have testified, and many more are ready to testify, that no such absolute immunity is known to them. As this is simply a question of fact, it must be decided, as in other cases of evidence, by the number and credibility of the witnesses; and, as the case has already been heard and decided, the number, on the one side, being large, clear, and consistent, and, on the other, few, confused, and equivocating, we may dismiss it at once, and for ever.

Mr. Fraser says, in pointing out the difference between what he calls the endemic and epidemic fevers of

Gibraltar, the bilious remittent and Bulam of Mr. Pym, that, in the former, there is little delirium. We are not prepared to question the accuracy of this assertion, as applied to Gibraltar; but, if it is meant of remittent fever generally, there can be no difficulty in pronouncing it incorrect. Among other records, let Mr. Fraser read Dr. Johnson's Work on Tropical Diseases, and he will find that fierce delirium is a common symptom of what is usually called bilious remittent fever. If this be the case in remittent fever elsewhere, and every body acquainted with the disease knows that it is the case, it may be so on some occasions at Gibraltar, and what then becomes of the diagnostic?

Further, in attempting to mark the two forms of fever, Mr. Fraser assures us that the endemic is "never infectious nor contagious," and that the "epidemic is obviously infectious." This reminds us of a notable specimen of induction in the "Elements of Medical Logic," amounting to this: The difference between the contagious and noncontagious fevers of the West Indies is to be known, among other things, by the fact one is contagious and the other not contagious. When men propose to teach us, we reasonably look for something by which we may be made wiser, and we are simple enough to think that we acquire little knowledge by being told that a thing is so because it is so, whether the information be communicated by the senior physician to the king, or by the medical superintendent of quarantine at Gibraltar.

Thirdly. On the subject of treatment, Mr. Fraser is singularly meagre, vague, and unsatisfactory. The object of his information, as far as it goes, appears to be chiefly to guard against bloodletting. Thus, at page 32, we are told: "I stated that, in certain cases of little danger, the lancet might be used among the robust and sanguineous, especially during warm weather; but, in the majority of cases, and those of real danger, the lancet was perfectly inadmissible." Now this amounts to a virtual prohibition of bloodletting as a curative agent; for, if it only *may* be used, it is obvious that it also *may* be omitted, which, indeed, is probable enough in cases of little danger; but we are told that, "in cases of real danger, it is perfectly inadmissible." Prepared as we in some measure were, by the preceding parts of the pamphlet, for strange things, we confess that this declaration startled us; we were not prepared, in 1826, for such a sweeping prohibition of this most valuable remedy. Such positive rules and regulations constitute the besetting sin of medicine, especially as regards fever. How often, since the days of Sydenham, has bloodletting been every thing or nothing in the treatment? During one lustrum, we are taught that little else is necessary but the abstraction of blood: during another, that to remove blood is to destroy life: and this over and over again, in regular rotation, till the superficial observer is led to believe that the treatment of fever is a mere matter of arbitration, to be determined by the will of him who has sufficient address to lead a party. But the man who looks more deeply, finds that such systems, either of exclusion or inclusion, are radically and ruinously wrong, especially when applied to the use of bloodletting in fever. He knows that, on every occasion of prevailing fever, different cases differ so greatly in character, that not only must different remedies be employed, but that the very principles on which they are employed ought to be different. In one case, of the same epidemic, there will be excitement, in another, depression of animal power; to apply the same remedies, in the same manner, and with the same views, in both cases, would be to run counter to the first principles of reasonable therapeutics, and to do our best to resist the sanatory efforts of nature. In attempting to adjust the various means to the ends proposed, no one is so important as the appropriate employment of bloodletting; and the man who does this, best performs the most important part in the treatment of fever. It is not enough to do this well, without doing other things, but it is assuredly one, and the first, of the essentials. Few dangerous cases of fever occur, we venture to affirm, in which bloodletting, in some stage, and to a certain amount, is not only admissible, but necessary; and the man who discharges his duty best is he who most skilfully employs it, as to time, quantity, and manner. We feel strongly on this subject, and therefore express earnestly our hope that it may be made a matter of graver study than, as appears from the pamphlet before us, it seems in some quarters to be. Such arbitrary and aphoristic directions, as that which we are considering, might be suitable enough in a pamphlet on the management of teeth, but are altogether inconsistent, in their manner at least, with such important subjects as the treatment of fever; and we hope that, if Mr. Fraser should write on the subject, and retain his opinion respecting bloodletting, he will condescend to give us some reason for it.

The principal of Mr. Fraser's ideas on treatment are given in the following words:

"Free and continued purgatives, chiefly calomel, colocynth, and gamboge, a strict antiphlogistic regimen, (generally speaking,) great care being taken not to overcharge the stomach; at the same time, abundance of broths, or diluents, were at command, together with warm baths, general and topical. Wine, soda-water, and porter, were freely used in convalescence. In fine, good nursing, the constant and apt exhibition of all those comforts so generally attainable in British practice, were found essential to the cure; the most expensive wines were occasionally issued, and the caprices of the sick indulged; pure and cool air was indispensable; warm lavations and baths were acceptable, but cold affusion was dangerous, if not mortal, and it was uniformly dreaded by the patients." (P. 31.)

The only remark we shall offer on this paragraph, containing the marrow of Mr. Fraser's therapeutical directions, is to suggest to the author, that it might not be amiss to take another look at Robert Jackson's book on febrile disease.

We can afford to give only one more specimen of Mr. Fraser's manner of instructing the ignorant, with which we shall take leave of these sybilline leaves:

"Cities escape when rigid enforcements seem to ward, and the wisdom of regulations is then lauded; but it has been asked, who, that has considered the illimitable range, the seemingly opposing facts, and the still undiminished mortality of epidemics, would consider himself qualified to decide that any virtual negligence, or any specific act, could deserve a fatal 'ostracism;' or that the voice or exertions of an individual had, in excluding pestilence, ever 'merited a mural crown.'" (P. 14.)

---

*Observations on the Nature and Treatment of Cholera; and on the Pathology of Mucous Membranes.* By Alex. Turnbull Christie, M.D. Madras Medical Establishment; and lately in Medical Charge of the Civil Department in the Southern Mahratta Country.—8vo. pp. 137. Edinburgh: Maclachlan and Stewart, 1828.

The author of this treatise states that he had considerable experience in the treatment of epidemic cholera during the years 1823, 24, 25, and 26, among the military in different parts of Madras, and particularly at Darwar, where even the secluded inmates of the jail were not safe from its fatal influence: and, as he was allowed to inspect the bodies of such of the prisoners as fell victims to the disease, he enjoyed an advantage of which, owing to the religious prejudices of the natives, the medical practitioner in India is generally deprived.

Dr. Christie does not profess to give a complete history of cholera, but, "by associating the observations of others with his own, he proposes to investigate its pathology, and endeavour to explain its symptoms, and the mode of action of the various remedies which have been employed for its cure."

It appears that the first cases of cholera which the author was enabled to investigate convinced him that the received opinions relating to its pathology were all more or less incorrect, and that the principal seat of the disease was in the whole mucous system; and, with the laudable desire of being better prepared to distinguish the precise effects of cholera on the mucous membranes, he instituted experiments on some of the lower animals; a few of which experiments are prefixed to this work, together with short remarks on the general pathology of mucous membranes. The latter commences thus:

"Inflammation, while it is the most frequent, appears also to be the most simple, morbid condition to which the various textures of the body are liable; and, in almost every texture, disease, however much it may vary in its progress and termination, is, with few exceptions, ushered in by inflammation.

"I will endeavour to show that the mucous system affords a remarkable exception to this general rule; for, in addition to inflammation, it is liable to another simple morbid affection, viz. catarrh, which often occurs alone, without being accompanied or having been preceded by inflammation. I conceive, then, that mucous membranes are liable to two distinct kinds of diseased action, viz. inflammation, evinced by one or more of the following signs, viz. increased heat, pain, redness, and swelling; and catarrh, characterised by the secretion of the membranes being depraved and increased in quantity." (P. 7.)

And,

"It has been stated as a general law, by several eminent authors, that inflammation of muccus membranes is accompanied by increased mucous secretion; and pathologists almost invariably attribute catarrh to an inflammation of the mucous membrane in which it occurs. This, I apprehend, is far from being correct; for are there not numerous examples of inflammation of a mucous membrane without increased secretion, and of catarrh without inflammation? We have examples of the former in ophthalmia, inflammatory sore throat, some cases of gastritis, and perhaps also of enteritis: of the latter, in a few cases of common catarrh, in diarrhœa, and also (as I shall have occasion to show in a future part of this essay) in Indian cholera." (P. 9.)

If we correctly apprehend the author, it appears to us that he here affirms much more than he can prove, or otherwise he ushers in with great importance as new, what has never been doubted: for surely there is no novelty in the opinion that inflammation is not *always* attended with increased mucous or other secretion, or that increased mucous secretion is not necessarily the effect of inflammation. On the other hand, it is evidently opposed both to reason and to experience to deny that inflammation is compatible with increased secretion; since if, as is generally believed, the matter secreted is in all instances derived from the blood, and if it is also true that inflamed organs contain, *cæteris paribus*, the greatest quantity of blood, then it is rational to conclude that one of the effects of moderate inflammation in mucous membranes would be an augmentation of their secretions; and daily practice tends to confirm us in this belief, since we are constantly meeting with patients who, with all the acknowledged signs of inflammation, have also increased secretion. But it may be asked whether it is logical to allow that to be sometimes an effect of inflammation which is not always so, especially when we admit that the same effect may arise from a different cause? We reply, that this is not more extraordinary than the acknowledged tendency of excessive inflammation of muscular parts to prevent the union of wounds, although that union is effected by what is called adhesive inflammation. That one degree of inflammation should diminish secretion, while another increases it, is, in fact, not more wonderful than that sleep should be promoted by one degree of fatigue, but prevented by another.

On this part of his work the author certainly does not tempt us to dwell, and we shall quote only one more passage from his "general remarks on the pathology of mucous membranes:"

"It is an important law of the animal economy, that there is always a determination of blood towards a part whose action is increased. In catarrh, the action of the excretory vessels of a mucous membrane is increased; a determination of blood, therefore, takes place towards them; and there is a consequent diminution of blood towards the surface. The size of the pulse, and heat of the skin, are thereby necessarily diminished. These are frequently referred to debility; but such an explanation is plainly inadmissible; for, in these cases, it is not the action of the vessels, but only the quantity of the blood circulating through them, which is diminished, whereby their caliber becomes contracted. For the confirmation of this view of the subject, we have only to appeal to facts. Great venous congestion is always found in the viscera of the thorax and abdomen of those who have died of catarrhal affections. Were the smallness of the pulse, in these diseases, owing to debility, we might expect that this smallness should occur, and that the natural fulness should return gradually; but we invariably find that the size of the pulse is very rapidly diminished when the secretion of the gastro-enteric mucous membrane is increased, and as rapidly restored to its natural condition upon the secretion being checked. Hence it is clear that the smallness of the pulse is owing to the blood having been withdrawn from the surface." (P. 16.)

This is, indeed, a singularly gratuitous assertion. What can be supposed to make the radial artery small and contracted, unless debility produce that effect? for surely this artery cannot be deemed an insignificant vessel. And, unless from debility, (no matter how produced,) why are the pulsations of the heart itself enfeebled, and the general powers of the system so amazingly reduced?

The author's experiments on dogs do not appear greatly calculated to elucidate or discover truths of any moment. He details eight cases in which he tried the effects, produced on the mucous membrane of the stomach, &c. of emetic tartar, corrosive sublimate, calomel, and opium, given separately, and in various proportions, to different dogs.

It is very proper that men should make up their minds upon every point of practical importance; and where simple analogy and induction on the experiments and reasonings of others are insufficient to remove doubt and to produce conviction, it is highly commendable to institute experiments calculated to terminate indecision, diminish the labours of research, and finally elicit valuable truths. While, however, we are willing to commend the honesty and prudence which forbid the admission of any thing of moment in medicine uncanvassed and unproved, we ought not to pass uncensured that ill-directed zeal which inundates the press with speculations founded upon experiment, which can as little enliven the leisure of the curious as instruct the study of the industrious. We are sorry to find that the main conclusion to which Dr. Christie is led by his experiments presents no very important form, when expressed in his own terms. The first clause contains what could not be doubted; the truth of the second was long since ascertained. But we quote the passage:

"Some medicines produce an inflammatory, others a catarrhal action, in mucous membranes; and a long-continued action of certain medicines produces the former, while a short-continued action of the same medicines produces the latter effect." (P. 42.)

The author, in his remarks on calomel, says,

"Its usual action on a mucous membrane is to excite its secretion; and in this case it renders the membrane white. If, however, its action be continued long on one spot, it gives rise to inflammation. This has been frequently observed in cholera; spots of inflammation having been observed in those parts of the mucous membrane of the stomach to which the calomel adhered." (P. 31.)

We are aware that Mr. ANNESLEY entertains the same opinion as Dr. C. respecting the *blanching* effect of calomel; but that it really possesses this property, is neither proved nor easy of demonstration: for, if M. BILLARD deserve any confidence, there is great reason to believe that the colour of the mucous membrane of the stomach and intestines is naturally white, excepting during the period of digestion, when it exhibits a slightly red tint.

*That the long-continued action of calomel on one spot* sometimes gives rise to inflammation, may indeed be true; but to think that this is always the case, would be very incorrect. In his interesting little work on Epidemic Cholera, Mr. BOYLE, on the contrary, relates that "fatal cases but too frequently occur where calomel is rejected as fast as it is given; or, *if retained*, is found, on examination after death, to have insinuated itself between the rugæ of the stomach, perfect in appearance, and without having had any effect whatever." (P. 56.)

Dr. C. asserts that

"There is no direct sympathy between the skin and liver; and the action of the liver and many other glands is much influenced by the condition of the mucous membrane upon which their excretory ducts open." (P. 42.)

Few, perhaps, will feel disposed to dispute the truth of the latter clause of this quotation; but we think the greater number of those who have witnessed the usual manifestations of hepatic disturbance, especially in tropical climates, will not readily admit the first. His arguments in favor of this opinion are hardly specious, and certainly not cogent; and we have too strong a recollection of the harsh and unnatural feel of the skin in dysentery, and particularly in chronic hepatitis of long standing, and of the never-failing indication of cure or amendment which the improved appearance and renewed functions of the skin so proverbially furnish in these diseases: we have too strong a recollection of these facts, to be able to adopt the author's notions, merely because an abundance of vitiated bile is passed in fevers when the skin continues parched and dry; or, on the contrary, because the skin is sometimes inundated with perspiration when the liver is apparently torpid and inert. This ought not to be considered a state, but a momentary struggle of the constitution, which, in the attempt to relieve itself from the oppressive influence of some deleterious cause, transgresses the laws of order and sympathy, and becomes the sport of diseased and irregular action: and, consequently, these morbid tendencies must not be cited as subversive of a theory supported by numerous observations and sound reasoning. We know that objections have been started against the theory of the "cutaneo-hepatic sympathy," but we are persuaded that it cannot easily be proved to be erroneous and unfounded.

The author attributes cholera not to inflammation, but to diseased action of the mucous membranes; and his main reason for doing so is founded upon a belief that the disease always leaves traces of its operation in some part of that system, while other organs and systems are only occasionally affected.

"In all the dissections I have made, the following appearances have been present: A whitish, opake, viscid substance was found adhering to the surface of some portions of the mucous membranes; and in many cases it was so abundant in the intestines as completely to fill parts of them of a greater or smaller extent. The stomach, and portions of the intestines, were filled with a transparent or turbid serous fluid; and frequently the viscid matter mentioned above was found intimately mixed with the serous fluid, or floating in it in the form of flakes. The mucous membranes (except when inflamed) had an unnatural whiteness, were frequently soft and pulpy, and in general (eapecially in the stomach and small intestines) could be easily detached by scraping, in the form of a thick pulp, from the subjacent coat. These appearances were sometimes more or less partial; but some of them were generally found throughout the whole extent of the alimentary canal. They extended, in some cases, to the mucous membrane of the bladder and ureters; and were found, in two or three instances, in the pulmonary mucous membrane." (P. 46.)

"The morbid appearances that have been found next in frequency to those already mentioned, are venous congestion in the viscera, particularly in those of the abdomen; dark-coloured blood in the veins, and sometimes in the left side of the heart; and inflammation in some part of the mucous membranes. I have generally found inflammation (when present at all) confined to the pyloric extremity of the stomach and small intestines. I have also met with many cases in which no inflammation could be detected." (P. 48.)

Many respectable names may, however, be cited, who have not constantly found organic disease of the primæ viæ in those who die of cholera, and who believe that, when such alteration of structure has occurred, it ought to be considered an effect and not the cause of the disease: they have also considered the cerebral affections the primary and most fatal. "In many cases the purging and vomiting have not been very violent, and people have suddenly become giddy, fallen down, and, after one or two slight efforts to vomit, have expired within a few minutes; and almost all who have been attacked have had some giddiness and pain of the head, a tendency to stupor, and have often become a little deaf. In two cases which I have seen, the jaw became locked for a time, but soon relaxed." (Dr. Alexander Gordon, *Bombay Report.*) The same gentleman mentions also a well-marked case of cholera, in which the *abdominal viscera evinced no trace of disease*, though the brain presented many morbid appearances. Indeed, it would be difficult, on Dr. Christie's theory, to account for the unusually rapid fatality of this disease which is observed in some instances.

Whoever is not totally unacquainted with the history of cholera, and the many speculations to which it has given rise, will perceive that Dr. C. pretends to no originality respecting it, besides that of referring the disease to a morbid condition, not merely of the mucous membrane of the stomach and intestines, but also of the whole mucous system: and on this point, he himself informs us, that he "has had it in his power only two or three times" to verify his opinion as far as it is peculiarly his own.

The author's remedies for cholera are rather numerous, but do not differ from those usually employed for its cure, viz. bloodletting, blisters, and sinapisms, hot sand, fumigations, frictions, calomel, opium, alcohol, ether, &c.; on the merits of each of which he separately discourses; but on the treatment of the disease we shall be content with quoting one of his general remarks:

"In the catarrhal cholera there will always be two principal indications of cure, viz. to remove the diseased action of the mucous membranes, and to restore the circulation of the blood towards the surface. The first will always be present; the second only after the disease has made some progress, and in all severe cases. But, in order to effect these indications, we shall require to employ different means under different circumstances, and to vary our remedies according as certain symptoms predominate or are awanting. We cannot expect, therefore, to discover any remedy or specific that will be applicable in all cases; and it is clear that there is just as much necessity for a practitioner to exercise his judgment in treating this as in treating any other disease in the whole range of the nosology." (P. 100.)

Dr. Christie certainly manifests considerable industry and much professional information; yet we fear he has brought his theory respecting cholera prematurely before the public, since it is not sufficiently supported by unequivocal facts. Though his remarks are sometimes judicious and interesting, yet, as we have already endeavoured to show, his conclusions do not always appear logical; and he occasionally takes great pains to establish points, concerning which, we believe, scarcely a doubt is entertained.

